# Motional narrowing, ballistic transport, and trapping of room-temperature exciton polaritons in an atomically-thin semiconductor

**DOI:** 10.1038/s41467-021-25656-7

**Published:** 2021-09-10

**Authors:** M. Wurdack, E. Estrecho, S. Todd, T. Yun, M. Pieczarka, S. K. Earl, J. A. Davis, C. Schneider, A. G. Truscott, E. A. Ostrovskaya

**Affiliations:** 1 ARC Centre of Excellence in Future Low-Energy Electronics Technologies and Nonlinear Physics Centre, Research School of Physics, The Australian National University, Canberra, ACT Australia; 2 Department of Experimental Physics, Wrocław University of Science and Technology, Wrocław, Poland; 3 ARC Centre of Excellence in Future Low-Energy Electronics Technologies and Centre for Quantum and Optical Science, Swinburne University of Technology, Victoria, Australia; 4 Institut für Physik, Carl von Ossietzky Universität Oldenburg, Oldenburg, Germany; 5 Laser Physics Centre, Research School of Physics, The Australian National University, Canberra, ACT Australia

**Keywords:** Polaritons, Two-dimensional materials

## Abstract

Monolayer transition metal dichalcogenide crystals (TMDCs) hold great promise for semiconductor optoelectronics because their bound electron-hole pairs (excitons) are stable at room temperature and interact strongly with light. When TMDCs are embedded in an optical microcavity, excitons can hybridise with cavity photons to form exciton polaritons, which inherit useful properties from their constituents. The ability to manipulate and trap polaritons on a microchip is critical for applications. Here, we create a non-trivial potential landscape for polaritons in monolayer WS_2_, and demonstrate their trapping and ballistic propagation across tens of micrometers. We show that the effects of dielectric disorder, which restrict the diffusion of WS_2_ excitons and broaden their spectral resonance, are dramatically reduced for polaritons, leading to motional narrowing and preserved partial coherence. Linewidth narrowing and coherence are further enhanced in the trap. Our results demonstrate the possibility of long-range dissipationless transport and efficient trapping of TMDC polaritons in ambient conditions.

## Abstract (web summary)

Room-temperature exciton polaritons in a monolayer WS_2_ are shown to display strong motional narrowing of the linewidth and enhanced first-order coherence. They can propagate for tens of micrometers while maintaining partial coherence, and display signatures of ballistic (dissipationless) transport.

## Introduction

Exciton polaritons (polaritons therein) are bosonic quasi-particles consisting of bound excitons and confined photons, which can form in optical microcavities with embedded direct bandgap semiconductors^1,2^ in the strong exciton-photon coupling regime. They inherit large group velocities from their photonic component, and interact due to their excitonic component, which enables them to display collective quantum phenomena^3–7^ in a solid state. A roadmap of polariton-based optoelectronic devices^8^ suggests multiple applications, including ultra-low threshold lasers^9^ and non-traditional computing architectures^10^. Proof-of principle demonstrations of these applications often rely on potential landscape engineering for the polaritons, e.g, by lithographic patterning, which is a well-established, advanced technology for epitaxially grown GaAs-based microcavities^11^. However, due to the low exciton binding energies in III–V semiconductors, their operation is limited to cryogenic temperatures. Although polariton condensation and trapping in engineered potential landscapes at room temperature were demonstrated by utilising semiconductors with large exciton binding energies^12–21^, the search for the optimal polaritonic material platforms that combine stability of the samples and low disorder continues^8^.

Recently, atomically thin, two-dimensional (2D) crystals of transition metal dichalcogenides (TMDCs) have emerged as very promising candidates for room-temperature polaritonics due to the large exciton binding energies and strong light-matter interactions^22,23^. Striking properties of TMDC polaritons^24^, such as the spin-valley Hall effect^25,26^, the formation of electrically charged polaron polaritons^27^, and signatures of bosonic condensation^28–30^ were explored in these systems.

**Fig. 1:**
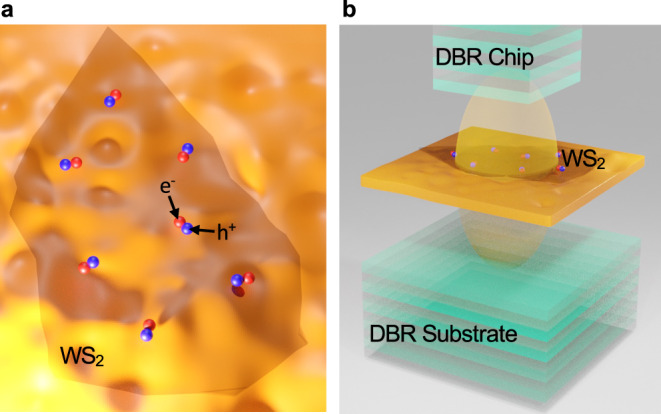
Excitons and polaritons in a disordered environment. **a** Schematic picture of monolayer WS_2_ hosting bound electron-hole pairs (excitons) placed on a substrate with substantial dielectric disorder with the spatial scale comparable with the exciton size^32^. The electrons and holes are represented by red (e^−^) and blue (h^+^) balls, respectively. **b** Hybridisation of excitons and photons, leading to formation of polaritons in an all-dielectric high-Q optical microcavity, reduces the effect of dielectric disorder^33^.

Here, we demonstrate room-temperature WS_2_ polaritons under non-resonant continuous-wave (cw) optical excitation in a high-quality all-dielectric monolithic microcavity with a non-trivial potential landscape. This potential landscape allows us to investigate both free and trapped WS_2_ polaritons in the “thermal” regime, below the onset of bosonic condensation. Excitons in monolayer WS_2_ were previously shown to be profoundly affected by dielectric disorder in the environment (Fig. 1a), which causes inhomogeneous linewidth broadening^31^ and reduction of the exciton diffusion coefficient due to scattering^32^. In contrast, we show that WS_2_ excitons strongly coupled to the cavity photons (Fig. 1b) exhibit dramatic motional narrowing^33^ of the inhomogeneously broadened linewidth, enhanced partial first-order coherence and long-range transport over tens of micrometers. Moreover, we find that polaritons propagating away from the excitation spot can conserve their total (potential and kinetic) energy, which points to the low-energy dissipation due to inelastic scattering with phonons and other carriers^7^. Thus, the polaritons exhibit strong signatures of ballistic transport at room temperature, with the propagation distances mainly limited by the radiative lifetime^34^. Long-range transport of polaritons enables their trapping in a quasi-1D potential well, even when the excitation spot is located tens of micrometers away from the trap.

## RESULTS

The all-dielectric monolithic microcavity investigated in this work was fabricated with the flip-chip approach^26,35^, by transferring a small piece of a dielectric Bragg reflector (DBR) from a polypropylene carbonate (PPC) film onto a DBR substrate at the position of a mechanically exfoliated WS_2_ monolayer (see Methods section and Supplementary Fig. 1 for details). This fabrication process fully maintains the excitonic properties of monolayer WS_2_ since it does not involve any direct material deposition on top of the monolayer, which can cause strong exciton quenching^36–38^.

### Polaritons in a non-trivial potential landscape

**Fig. 2:**
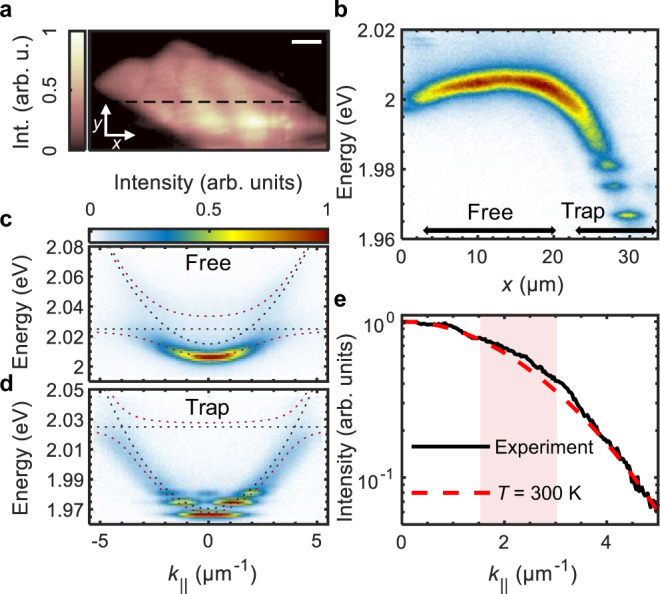
Potential landscape and polariton dispersion. **a** PL map of the monolayer region inside the microcavity, where *x* and *y* mark the directions along and perpendicular to the spectrometer slit, respectively. Scale bar = 5 μm. **b** PL spectrum at approximately zero in-plane momentum (*k*_∣∣_ ≈ 0) across the dashed line in panel **a**. **c**, **d** Polariton dispersion extracted from the angle-resolved photoluminescence spectra of the sample (**c**) in the “planar” region with a weak potential gradient (marked “Free” in **b**) and **d** in the trap region (marked “Trap” in **b**). The flat (parabolic) black dotted lines correspond to the exciton (cavity photon) energies and the upper (lower) red dotted lines are the fitted upper (lower) polariton branches with the Rabi splitting 2ℏΩ ≈ 25 meV. **e** Intensity profile of the PL spectrum in **c** with the theoretical intensity profile of a fully thermalised polariton gas at *T* = 300 K. The bottleneck region of the free polariton dispersion (between *k*_∣∣_ ≈ 1.5 μm^−1^ and *k*_∣∣_ ≈ 3 μm^−1^) is shaded.

When the microcavity is excited by a continuous wave (cw) frequency doubled ND:YAG laser through a transmission maximum of the DBR mirror at *λ* = 532nm, the intensity map of the photoluminescence (PL) at the position of the monolayer shows strong polariton emission (see Fig. 2a). Measuring the polariton emission spectrum along the dashed line at the angle of approximately zero incidence (see Methods section) allows us to estimate the profile of the potential landscape^34,39^ for polaritons corresponding to zero kinetic energy (in-plane momentum *k*_∣∣_ ≈ 0). This measurement reveals the non-trivial shape (see Fig. 2b) of the potential caused by strong variation of the detuning between the cavity photon energy, *E*_C_, and the exciton resonance, *E*_X_. This variation is likely caused by an air gap between the DBR chip and the DBR substrate, which leads to a local modification of the cavity length. As a result, the polariton ground state (energy at *k*_∣∣_ = 0) considerably varies at positions *x* > 20 μm, and an effective trap for polaritons is formed at *x* = 30 μm. The trap is a quasi-1D potential well strongly elongated in the *y*-direction perpendicular to the measurement direction, *x* (see Supplementary Fig. 2). The angle- (momentum-)resolved PL spectra (dispersion) in the “planar” region with a weak potential gradient (*x* < 20 μm) (Fig. 2c) and in the effective trap (Fig. 2d) are well fitted with the lower (LP) and upper (UP) polariton branches ${E}_{{{{{{{{\rm{LP/UP}}}}}}}}}=\frac{1}{2}[{E}_{{{{{{{{\rm{X}}}}}}}}}+{E}_{{{{{{{{\rm{C}}}}}}}}}\pm \sqrt{{(2\hslash {{\Omega }})}^{2}+{\delta }^{2}}]$, where *δ* = *E*_X_ − *E*_C_ is the exciton-photon energy detuning, and 2*ℏ*Ω is the Rabi splitting^1,6^. Therefore, the sample operates in the strong exciton-photon coupling regime at room temperature across its whole area, within a large range of detunings *δ* ∈ [10, 60] meV. The detuning defines the excitonic fraction of the polariton through the excitonic Hopfield coefficient^6^, which takes values ∣*X*^F^∣^2^ ≈ 0.3 and ∣*X*^T^∣^2^ ≈ 0.05 for the free and trapped polaritons, respectively (see Supplementary Fig. 3).

The occupation numbers of the momentum states of free polaritons (at *x* = 16 μm), with the dispersion shown in Fig. 2c, are reflected in the intensity of the PL profile and can be well described with a model of a fully thermalised polariton gas^40^ (see Supplementary Fig. 4) at room temperature, *T* = 300 K (see Fig. 2e). In the well-established model for the polariton energy relaxation under non-resonant excitation^7,14,41–44,45^, polaritons reduce their kinetic energy by inelastic scattering with phonons, excitons, and other polaritons. While the effective inter-particle interactions in our structure are weak due to the large binding energies and small Bohr radii of excitons^46^, the observed thermalisation implies thermal equilibrium of the polariton gas with the environment and efficient polariton-phonon interactions at room temperature. The slight departure towards the higher occupation numbers in Fig. 2e occurs around the inflection point of the polariton dispersion (see Supplementary Fig. 3), referred to as the relaxation bottleneck^7,47,48^, where the density of states reduces dramatically, the effective mass changes sign, and interaction with phonons becomes less efficient.

Quantisation of the trapped polariton spectrum is highly pronounced in Fig. 2d because the lateral size of the trap in the *x*-direction (a few micrometers) is comparable to the thermal de Broglie wavelength of polaritons^49^, ${\lambda }_{{{{{{{{\rm{th}}}}}}}}}=\sqrt{2\pi {\hslash }^{2}{\left({m}_{{{{{{{{\rm{eff}}}}}}}}}{k}_{B}T\right)}^{-1}}$^50^, where *m*_eff_ is the polariton effective mass extracted from the dispersion fits in Fig. 2c, d. At *T* = 300 K, we obtain for the free and trapped polaritons $\lambda _{{{{{\rm{th}}}}}}^{{{{{\rm{F}}}}}}=(1.01\pm 0.05)\,\upmu {{\rm{m}}}$ and $\lambda _{{{{{\rm{th}}}}}}^{{{{{\rm{T}}}}}}=(1.18\pm 0.05)\,\upmu {{\rm{m}}}$, respectively. The ground, first, and second excited states in the trap are clearly occupied, and the energies of these states fit well to the simulation results (see Supplementary Fig. 5).

### Motional narrowing of polariton linewidth

**Fig. 3:**
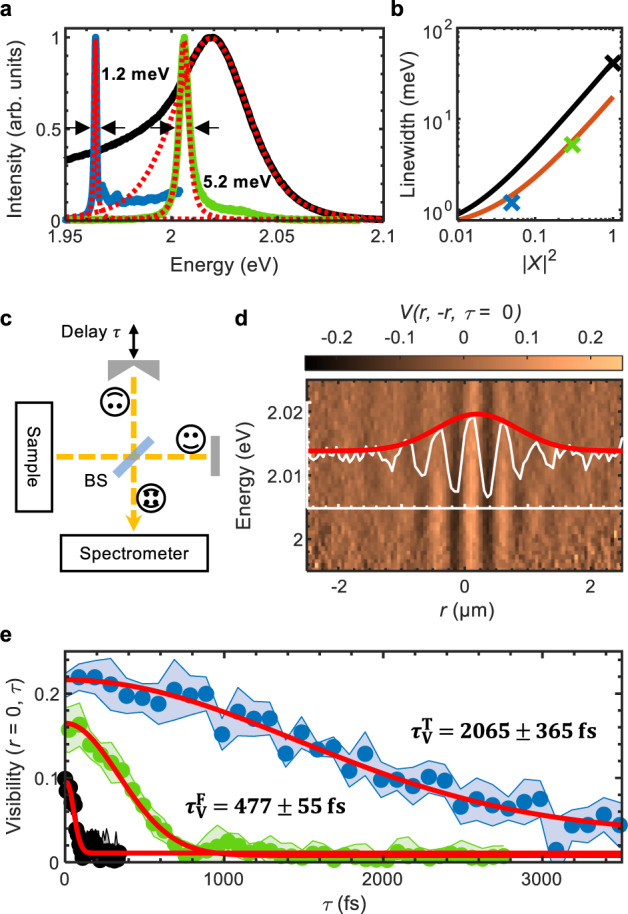
Linewidth and coherence measurements. **a** PL spectra at *k*_∣∣_≈  0 of the free polaritons (green), trapped polaritons (blue), and of WS_2_ excitons in the bare monolayer on the same substrate (black). The WS_2_ exciton spectrum and the polariton ground states were fitted with a Voigt line profile (red dots). **b** Theoretical polariton linewidth as a function of excitonic Hopfield coefficient, based on the WS_2_ exciton linewidth (see text) and a cavity mode with the quality factor *Q* ≈ 3000 (see Methods section), with (black) and without (orange) contribution of inhomogeneous exciton linewidth broadening. Crosses mark the linewidths of the exciton (black) and polariton (green and blue) emissions from the data in panel **a**. **c** Schematic of the experimental setup of the modified Michelson interferometer (see Methods section). **d** Normalised, spectrally resolved interferogram of the free polaritons at zero time delay (see Methods section) superimposed with the interference fringes at their lowest energy *E* = 2.004 eV in the range [−0.2, 0.2] (white) and the corresponding fringe visibility (red). **e** Time coherence measurements of the excitons (black data points), free polaritons (green data points), and trapped polaritons (blue data points) at their lowest energies, fitted with a second order exponential decay function (red lines). The extracted decay times for the fringe visibility are ${\tau }_{{{{{{{{\rm{V}}}}}}}}}^{{{{{{{{\rm{X}}}}}}}}}=(62\pm 15)\,{{{{{{{\rm{fs}}}}}}}}$ for the excitons, ${\tau }_{{{{{{{{\rm{V}}}}}}}}}^{{{{{{{{\rm{F}}}}}}}}}=(477\pm 55)\,{{{{{{{\rm{fs}}}}}}}}$ for the free polaritons, and ${\tau }_{{{{{{{{\rm{V}}}}}}}}}^{{{{{{{{\rm{T}}}}}}}}}=(2065\pm 365)\,{{{{{{{\rm{fs}}}}}}}}$ for the trapped polaritons. The shaded areas correspond to the uncertainties of the fitting procedure and represent a 95% confidence interval. For the measurements in **a**, **d**, **e**, the PL is collected at the position of the excitation spot.

To quantify the effect of the dielectric disorder on the WS_2_ polaritons at room temperature, we investigate the PL spectra of both free and trapped polaritons at *k*_∣∣_ ≈ 0 by employing filtering in *k*-space (see Methods section), and compare it to the PL spectrum of bare excitons in a WS_2_ monolayer on the same DBR substrate (see Fig. 3a). The high energy tail of the polariton emission arises due to the Boltzmann distribution of the thermalised polaritons, while the low energy tail of the bare exciton emission is mostly due to formation of charged excitons (e.g., trions) and excitons bound by defects. Disregarding the tails, the exciton and lower polaritons peaks are fitted with convoluted Lorentzian and Gaussian distributions (Voigt function), corresponding to the homogeneous and inhomogeneous linewidth broadening, respectively. The homogeneous broadening is mainly determined by the radiative decay of the excitons and exciton-phonon interactions^51^, and the inhomogeneous broadening is largely due to dielectric disorder on the substrate surface, which causes local fluctuations of the exciton binding energies and of the free carrier bandgap^31^. For the excitons, we extract a total linewidth of Δ*E*_X_ = (41.5 ± 0.5) meV, with the homogeneous linewidth broadening of ${{\Delta }}{E}_{{{{{{{{\rm{X}}}}}}}}}^{{{{{{{{\rm{H}}}}}}}}}=(17.5\pm 0.3)\,{{{{{{{\rm{meV}}}}}}}}$ and the inhomogeneous linewidth broadening of ${{\Delta }}{E}_{{{{{{{{\rm{X}}}}}}}}}^{{{{{{{{\rm{IH}}}}}}}}}=(31.3\pm 0.2)\,{{{{{{{\rm{meV}}}}}}}}$ within 95% confidence level. The homogeneous linewidth broadening corresponds to ${\gamma }_{{{{{{{{\rm{X}}}}}}}}}^{-1}=2\hslash /{{\Delta }}{E}_{{{{{{{{\rm{X}}}}}}}}}^{{{{{{{{\rm{H}}}}}}}}}=\left(75\pm 2\right)\,{{{{{{{\rm{fs}}}}}}}}$, which is in good agreement with the decoherence rate of excitons in a WS_2_ monolayer placed on a high-quality SiO_2_ substrate, as determined in the four-wave mixing experiments (see Supplementary Note and Supplementary Fig. 6).

Compared to the excitons, both free and trapped polaritons display much narrower linewidths (see Fig. 3a) of ${{\Delta }}{E}_{{{{{{{{\rm{F}}}}}}}}}=\left(5.2\pm 0.1\right)\,{{{{{{{\rm{meV}}}}}}}}$ and ${{\Delta }}{E}_{{{{{{{{\rm{T}}}}}}}}}=\left(1.20\pm 0.01\right)\,{{{{{{{\rm{meV}}}}}}}}$, respectively. The inhomogeneous broadening is ${{\Delta }}{E}_{{{{{{{{\rm{F}}}}}}}}}^{{{{{{{{\rm{IH}}}}}}}}}=(2.6\pm 0.1)\,{{{{{{{\rm{meV}}}}}}}}$ for free polaritons and is negligible for trapped polaritons, which represents a significant reduction compared to inhomogeneous broadening of excitons (${{\Delta }}{E}_{{{{{{{{\rm{X}}}}}}}}}^{{{{{{{{\rm{IH}}}}}}}}} \sim 31\,{{{{{{{\rm{meV}}}}}}}}$). Moreover, the linewidths of the free and trapped lower polaritons are substantially smaller than the theoretically calculated (see Methods section) polariton linewidth (Fig. 3b, black line). The theoretical and the measured linewidths agree well only when the inhomogeneous exciton broadening is completely eliminated from the calculation (Fig. 3b, orange line). This indicates that the effects causing inhomogeneous linewidth broadening are significantly reduced for the WS_2_ excitons strongly coupled to the cavity photons. This so-called motional narrowing is a well-known effect in quantum well microcavities^33,52,53^, and was recently observed at cryogenic temperatures for excitons in monolayer MoSe_2_ strongly coupled to cavity photons^54^ and to optical bound states in the continuum^55^. The motional narrowing occurs due to the size of polaritons, *λ*_th_, significantly exceeding the spatial scale of dielectric disorder-induced energy fluctuations^33^, with a sufficiently strong exciton-photon coupling^53^. The strong reduction of inhomogeneous broadening observed here for trapped and free polaritons at room temperature is an order of magnitude larger than ~1 meV motional narrowing previously observed in quantum well based semiconductor microcavities at cryogenic temperatures^33^.

### Extended range of partial macroscopic coherence

In addition to the inhomogeneous linewidth broadening, dielectric disorder causes rapid dephasing of WS_2_ excitons. Combined with decoherence caused by radiative losses and scattering with phonons and other carriers (see Supplementary Note), this leads to loss of the macroscopic phase coherence. To compare the timescales of macroscopic decoherence exhibited by the excitons and polaritons, we perform the coherence measurements with a modified Michelson interferometer (see Fig. 3c)^3^. In this configuration, overlapping the original image *I*_*o*_ with its flipped and time-delayed copy *I*_*f*_ causes interference fringes, which can be normalised as (see Methods section):

1 $$V(r,-r,\tau )=\left[{I}_{{{{{{{{\rm{t}}}}}}}}}-\left({I}_{{{{{{{{\rm{o}}}}}}}}}+{I}_{{{{{{{{\rm{f}}}}}}}}}\right)\right]{\left(2\sqrt{{I}_{{{{{{{{\rm{o}}}}}}}}}{I}_{{{{{{{{\rm{f}}}}}}}}}}\right)}^{-1},$$

where *r* is the distance from the axis of the retroreflector, *τ* is the time delay between the interferometer arms, and *I*_t_ is the total intensity at *r* and *τ* (see Methods). The envelope of *V*(*r*, − *r*, *τ*) (the visibility) is proportional to the absolute value of the first-order coherence function $\left|{g}^{(1)}(r,\tau )\right|$, and therefore a drop in visibility indicates a loss of the macroscopic coherence (see Methods section). For the coherence measurement, we remove the *k*-space filtering to avoid filter artefacts and employ a spectrometer to compare *V*(*r*, −*r*, *τ*) of the excitons, free polaritons and trapped polaritons in their lowest energy states. The normalised, spectrally resolved interference pattern of the free polaritons at zero delay *τ* = 0 is shown in Fig. 3d, together with *V*(*r*, −*r*, *τ* = 0) and its envelope as a measure for $\left|{g}^{(1)}(r,\tau =0)\right|$. The full width at half maximum ${{{{\rm{FWHM}}}}}(\left|{g}^{(1)}\right|)=\left(1.5\pm 0.1\right)\,\upmu m$ is much larger than the theoretically expected value^50^ for the thermal polariton gas ${\lambda }_{{{{{{{{\rm{th}}}}}}}}}\sqrt{{{{{{{\mathrm{ln}}}}}}}\,2/(4\pi )}\approx 0.24\,{{{{\mu m}}}}$. This indicates that the polaritons can maintain partial macroscopic coherence while rapidly expanding, likely as a result of diminished disorder-induced dephasing and weak inter-particle interactions.

The time coherence measurements are performed at *τ* ≠ 0. Figure 3e shows the envelopes of *V*(*r* = 0, *τ*) for the excitons, free polaritons, and trapped polaritons around their lowest energies, fitted with a second-order exponential decay function^56^. We define the time at which *V*(*r* = 0, *τ*) drops to 1/e as the decay time of the visibility *τ*_*V*_. While the absolute values of *τ*_*V*_ extracted from the data in Fig. 3e are most likely larger than the actual decay times of macroscopic coherence, due to the spatial resolution of our experimental setup^50^, their relative values scale with the relative linewidths of the excitons, free polaritons, and trapped polaritons as: ${{\Delta }}{E}_{{{{{{{{\rm{X}}}}}}}}}/{{\Delta }}{E}_{{{{{{{{\rm{F}}}}}}}}}={\tau }_{{{{{{{{\rm{V}}}}}}}}}^{{{{{{{{\rm{F}}}}}}}}}/{\tau }_{{{{{{{{\rm{V}}}}}}}}}^{{{{{{{{\rm{X}}}}}}}}}\approx 8$, and ${{\Delta }}{E}_{{{{{{{{\rm{X}}}}}}}}}/{{\Delta }}{E}_{{{{{{{{\rm{T}}}}}}}}}={\tau }_{{{{{{{{\rm{V}}}}}}}}}^{{{{{{{{\rm{T}}}}}}}}}/{\tau }_{{{{{{{{\rm{V}}}}}}}}}^{{{{{{{{\rm{X}}}}}}}}}\approx 33$, showing that the changes in total linewidths directly correlate with the decay of coherence. Hence, due to the motional narrowing of inhomogeneous linewidth, the macroscopic coherence of the WS_2_ polaritons is barely affected by dielectric disorder and is determined by their radiative lifetime and scattering processes contributing to the homogeneous linewidth broadening.

### Long-range polariton transport

**Fig. 4:**
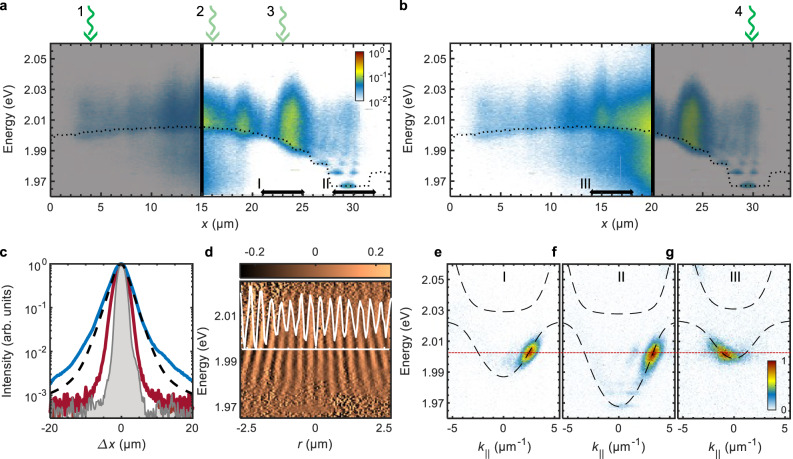
Long-range polariton transport. **a** Position-resolved PL spectrum of the polaritons excited in the shaded area at position 1, *x* = 4 μm (green arrow), and measured in the clear area at *x* > 15 μm. **b** Position-resolved PL spectrum of the polaritons excited in the shaded area at position 4, *x* = 30 μm (green arrow), and measured in the clear area at *x* < 20 μm. The outline of the polariton energy at *k* ≈ 0, obtained by extracting spectral peak position from the signal in Fig. 2b, is marked with a dotted black line. Green (1-4) and black (I-III) arrows mark excitation and detection positions for panels **c**–**g**, respectively. **c** Real-space profile of the laser excitation spot (grey), and the corresponding PL profiles of the exciton emission of a monolayer WS_2_ on the DBR substrate (red), and polariton emission of the microcavity (blue), when the sample is excited at position 2. The black dashed line is the modelled PL profile of the thermal polariton gas at *T* = 300 K (see Supplementary Fig. 4). **d** Normalised, spectrally resolved interferogram of the travelling polaritons in the region II, when the sample is excited at position 3. The unit of the colour bar is fringe visibility. The superimposed line plot (white) shows the normalised interference fringes at *E* = 1.995 eV in the range [ − 0.2, 0.2]. **e**, **f** Normalised momentum-resolved PL spectra in the detection regions (**e**) I and (**f**) II, when the sample is excited at position 2. **g** Normalised momentum-resolved PL spectrum in the detection region III, when the sample is excited at position 4. The spectra in **e**–**g** are measured with different spectrometer settings and acquisition times and then normalised to compare the positions of the travelling wave packets in the (*k*_∣∣_,E) parameter space rather than their intensities. The polariton dispersions (black dashed lines) in panels **e**, **g** are calculated based on the measured polariton energy at *k*_∣∣_ ≈ 0 (Fig. 2b), the exciton energy, the Rabi splitting, and the effective mass of the cavity photon at positions I and III. The actual dispersion at position I may slightly deviate from this estimate due to variation of the cavity thickness, which leads to ~4 meV uncertainty in the kinetic energy of the propagating polaritons in **e**. The dispersion in panel **f** corresponds to the measured dispersion branches in Fig. 2d. The red dashed line in **e**-**g** marks *E* = 2.003 eV.

Scattering of bare WS_2_ excitons on disorder also strongly reduces their diffusion coefficient and limits their mobility^32^. In order to contrast the propagation of the WS_2_ polaritons with excitons, we collect the PL at the positions away from the excitation spot (see Methods section). The clear area in Fig. 4a shows the real-space resolved PL spectrum along the dashed line in Fig. 2a, when exciting the sample at *x* = 4 μm (position 1) and collecting the PL at positions *x* > 15 μm. Conversely, the clear area in Fig. 4b shows the real-space resolved PL spectrum when the sample is excited at the opposite side of the sample, at *x* = 30 μm (position 4) and the PL is collected at positions *x* < 20 μm. Strikingly, regardless of the excitation position, the polaritons travel across the whole monolayer area, both up and down the potential gradient shown in Fig. 2b, which illustrates the long-range propagation of the WS_2_ polaritons exceeding the mean diffusion length of WS_2_ excitons (360 nm^57^) by orders of magnitude.

By measuring the real-space PL profile when exciting either the microcavity or the bare monolayer WS_2_ with a focused laser spot (at position 2 in Fig. 4a for the microcavity sample), we can quantitatively compare the WS_2_ polariton transport with the diffusion of WS_2_ exciton. Figure 4c shows the emission profiles of the microcavity polaritons, the monolayer excitons, and the laser spot on logarithmic scales. It is clear that the line shape of the polariton PL strongly differs from the approximately Gaussian line profile of the laser and the monolayer PL. This is caused by the Boltzmann distribution of the thermalised polariton gas^40^, in which the upper polariton branch and the lower polariton branch at higher *k*_∣∣_ values are well occupied at room temperature (see Fig. 2e). The corresponding modelled PL profile of the polariton gas at *T* = 300 K (black dashed line in Fig. 4c), expanding with the group velocity and lifetime corresponding to the relaxation bottleneck of the free polaritons (Fig. 2c), qualitatively reproduces the measured profile. The calculations for different temperatures show that the rapid expansion of the polariton gas from the pump spot is promoted by its high temperature (see Supplementary Fig. 4). The extent of the measured profile is larger than the calculated profile most likely due to the higher polariton occupation numbers in the bottleneck region compared to the fully thermalised polariton gas (see Fig. 2b). These results suggest that the bottleneck polaritons, which interact weakly with phonons and possess maximum group velocity (see Supplementary Fig. 3), strongly contribute to the polariton flow. While the exciton PL intensity drops to 1% at Δ*x* ≈ 5 μm, the polariton intensity reaches this level at Δ*x* ≈ 12 μm. For comparison, the laser excitation drops to this intensity level at Δ*x* ≈ 3 μm, and with a (sub)linear relationship between excitation intensity and PL intensity^58^ the transport length of the polaritons in this structure is at least five times larger compared to the excitons. The agreement between the model and experiment in Fig. 4c implies that the transport length of polaritons is mainly limited by their lifetime.

The spatial coherence of the travelling polaritons was investigated by exciting the structure at position 3 in Fig. 4a and measuring the interferogram of the travelling wave packet at position II (see Fig. 4d), i.e., in the trap region. The interference fringes, extracted by removing the Doppler effect, have a substantial spatial extent, and their visibility is similar to the maximum visibility of the interference pattern at the excitation spot (see Fig. 3d and Supplementary Fig. 8). The extent of the spatial coherence can be well explained by the high kinetic energy of the travelling wave packet, and the conservation of the magnitude of the fringe visibility is a direct result of the reduced dephasing and decoherence. This finding further confirms that disorder-induced effects are heavily reduced in the strongly light-matter coupled system. Moreover, the conservation of partial macroscopic coherence means that inelastic scattering processes are suppressed for the travelling polaritons, implying a strong contribution of the bottleneck polaritons to the polariton flow.

### Ballistic transport of polaritons

Finally, we analyse the dispersion of the propagating polaritons by collecting the angle-resolved PL spectra from small areas in the real space (see Methods section). In contrast to real space-resolved spectral imaging (Fig. 4a, b), in which the emitted PL is collected in all directions of the light cone, angle-resolved spectral imaging collects the PL signal limited to angles of incidences along the spectrometer slit direction. This allows us to characterise the momentum-resolved energy spectrum of the polaritons in one particular direction *x*, i.e. along the dashed line in Fig. 2a. First, we excite the sample at position 2 and measure PL at positions I and II in Fig. 4a. The spectra (Fig. 4e, f) show that the polaritons are travelling at a constant energy centred at *E* ≈ 2.003eV (red dashed line in Fig. 4e, f) along the potential gradient, and that their potential energy (with respect to the global minimum) is almost fully converted into kinetic energy (i.e., the energy at *k* measured with respect to the energy at *k* = 0). The constant energy of the propagating polaritons approximately coincides with the inflection point of the lower polariton dispersion, which marks the relaxation bottleneck. We attribute this apparent lack of the energy relaxation to weak inter-particle and polariton-phonon interactions at the relaxation bottleneck^7,46^, which results in the reduced energy dissipation in the system. Hence, room-temperature polaritons in WS_2_ can propagate ballistically over tens of micrometers.

Remarkably, when swapping the excitation and detection positions, i.e. exciting the polaritons at the position of the trap (position 4 in Fig. 4b), and measuring the angle-resolved PL spectra at position III in Fig. 4b, we find that the energy of the polaritons moving up the potential hill is approximately the same as the energy of the downhill flow, see Fig. 4g. This effect is also seen in the upper polariton branch, as shown in Supplementary Fig. 7. This uphill flow is due to the high-momenta thermalised polaritons excited in the trap region (Fig. 2d) with above-barrier kinetic energies. Without energy dissipation, these polaritons efficiently convert their high kinetic energy into potential energy while flowing uphill and populate the planar region of the sample, as observed in Fig. 4b. The absence of backscattering signal at the opposite momentum further confirms that scattering on disorder is negligible.

Despite the constant energy flow along the gradient (*x*-direction), clear energy relaxation and the resulting occupation of the low-energy trapped states is visible in the position-resolved spectral image (Fig. 4a, region II), which collects emission from polaritons travelling in all directions, including that orthogonal to the quasi-1D trap, i.e. direction *y* in Fig. 2a. Weak signatures of this relaxation are visible in Fig. 4f, but the signal is stronger for the emission not filtered along *x*. This indicates that the phonon-induced energy relaxation for room temperature polaritons is sufficient to drive the occupation of the lower energy states for polaritons accumulating in the trap. The trap is occupied by polaritons even with the excitation spot is located tens of micrometers away at the opposite side of the monolayer, i.e. at position 1 in Fig. 4a.

## Discussion

In summary, we have realised freely moving and trapped WS_2_ polaritons in a non-trivial potential landscape at room temperature. The pronounced motional linewidth narrowing and enhanced macroscopic coherence of the polaritons point to dramatic reduction of the effects of dielectric disorder, which strongly affect the bare exciton dynamics in monolayer TMDCs. The weak effective inter-particle and polariton-phonon interactions in the relaxation bottleneck enable the polaritons to travel across tens of micrometers with minimal energy dissipation, and to maintain their partial macroscopic coherence while propagating. These findings elucidate the characteristic features of the dynamics of WS_2_ polaritons at room temperature and the role of dielectric disorder in the TMDC systems strongly coupled to light. The demonstrated long-range ballistic flow and trapping of polaritons in the lowest energy states of a quasi-1D potential represent a significant step towards developing methods for manipulating and trapping polariton flow in TMDC-based polaritonic devices.

## Methods

### Sample fabrication

A DBR chip splintered off a DBR substrate was placed on top of a polypropylene-carbonate (PPC) film^35^, which was initially spin-coated on top of a PDMS stamp supported by a glass slide. The two halves of the SiO_2_* λ*/2-spacer were deposited by RF magnetron sputtering on top of the DBR chip and a DBR substrate, respectively, to ensure that the photonic field has its maximum at the centre of the microcavity. Further, a mechanically exfoliated monolayer WS_2_ was transferred on top of the DBR substrate. Finally, the cavity was mechanically assembled with a van der Waals stacking stage, at a temperature at which the DBR chip detaches from the PPC film (130 °C).

### Experimental setup

The photoluminescence spectra were measured with an in-house built optical setup, equipped with an array of lenses allowing for real-space (RS) and momentum-space (KS) imaging. The filtering in RS and KS were achieved with an edge-filter and an iris in the respective image planes. RS and KS imaging can be switched by flipping the lens, which images KS, in or out of the beam path. The RS and KS spectra were measured with a spectrometer equipped with a CCD-camera and different spectrometer gratings, with 150, 600, and 1200 lines per mm, allowing for energy resolutions down to 60μeV per pixel. For the coherence measurements, we implemented a modified Michelson interferometer, where one arm is equipped with a retroreflector^3^ that flips the image vertically. The output of the interferometer is fed onto the spectrometer and the interfering images are recorded using a CCD camera. The retroreflector arm is translated using a motorised stage to change the delay between the two arms.

### Polariton linewidth

To estimate the polariton linewidth, we calculate the theoretical coherence times in an ideal system for the excitons and for the microcavity photons based on their Gaussian and Lorentzian linewidth contributions^56^:

2 $${\tau }_{{{{{{{{\rm{X/C}}}}}}}}}^{{{{{{{{\rm{H}}}}}}}}}={\left(\pi {{\Delta }}{f}_{{{{{{{{\rm{X/C}}}}}}}}}^{{{{{{{{\rm{H}}}}}}}}}\right)}^{-1},{\tau }_{{{{{{{{\rm{X/C}}}}}}}}}^{{{{{{{{\rm{IH}}}}}}}}}=\sqrt{2{{{{{{\mathrm{ln}}}}}}}\,(2)}{\left(\sqrt{\pi }{{\Delta }}{f}_{{{{{{{{\rm{X/C}}}}}}}}}^{{{{{{{{\rm{IH}}}}}}}}}\right)}^{-1}.$$

Here Δ*f* = Δ*E*/*h*. In the strong coupling regime, the polariton coherence time is determined by the exciton and cavity photon coherence times weighted by the excitonic Hopfield coefficient^6^:

3 $${\tau }_{{{{{{{{\rm{P}}}}}}}}}^{{{{{{{{\rm{H/IH}}}}}}}}}({\left|X\right|}^{2})={\left({\left|X\right|}^{2}/{\tau }_{{{{{{{{\rm{X}}}}}}}}}^{{{{{{{{\rm{H/IH}}}}}}}}}+(1-{\left|X\right|}^{2})/{\tau }_{{{{{{{{\rm{C}}}}}}}}}^{{{{{{{{\rm{H/IH}}}}}}}}}\right)}^{-1}.$$

By using the formulas above, we deduct the theoretical values for inhomogenous and homogenous broadening from the theoretical coherence times and obtain for the total linewidth of the resulting Voigt line profiles:

4 $${{\Delta }}{f}_{{{{{{{{\rm{P}}}}}}}}}({\left|X\right|}^{2})=0.5346{{\Delta }}{f}_{{{{{{{{\rm{P}}}}}}}}}^{{{{{{{{\rm{H}}}}}}}}}+\sqrt{0.2166{{\Delta }}{{f}_{{{{{{{{\rm{P}}}}}}}}}^{{{{{{{{\rm{H}}}}}}}}}}^{2}+{{\Delta }}{{f}_{{{{{{{{\rm{P}}}}}}}}}^{{{{{{{{\rm{IH}}}}}}}}}}^{2}}.$$

Without inhomogeneous broadening, the polariton linewidth can be directly calculated as:

5 $${{\Delta }}E=| X{| }^{2}{{\Delta }}{E}_{{{{{{{{\rm{X}}}}}}}}}^{{{{{{{{\rm{H}}}}}}}}}+(1-| X{| }^{2}){{\Delta }}{E}_{{{{{{{{\rm{C}}}}}}}}},$$

where Δ*E*_C_ is the cavity photon linewidth for the microcavity with the quality factor *Q* ≈ 3000 (see Supplementary Fig. 9).

### Interference visibility

The interference image measured by our camera *I*_*tot*_ can be written as^59^:

6 $${I}_{{{{{{{{\rm{t}}}}}}}}}(r,\tau )={I}_{{{{{{{{\rm{o}}}}}}}}}(r)+{I}_{{{{{{{{\rm{f}}}}}}}}}(r)+2| {g}^{(1)}(r,\tau )| \sqrt{{I}_{{{{{{{{\rm{o}}}}}}}}}(r){I}_{f}(r)}\cos (\kappa r+\phi ),$$

where *κ* and *ϕ* correspond to the fringe frequency and relative phase, respectively. The normalised interferograms presented in this work are calculated using the formula:

7 $$V(r,-r,\tau )=\frac{{I}_{{{{{{{{\rm{t}}}}}}}}}(r)-\left({I}_{{{{{{{{\rm{o}}}}}}}}}(r)+{I}_{{{{{{{{\rm{f}}}}}}}}}(r)\right)}{2\sqrt{{I}_{{{{{{{{\rm{o}}}}}}}}}(r){I}_{{{{{{{{\rm{f}}}}}}}}}(r)}}.$$

The first-order coherence function is the envelope of the normalised interferogram, as given by:

8 $$| {g}^{(1)}(r,\tau )| \cos (\kappa r+\phi )=V(r,-r,\tau ).$$

## Supplementary information

Supplementary Information

## Data Availability

The data that support the findings of this study are available from the corresponding authors upon reasonable request.
