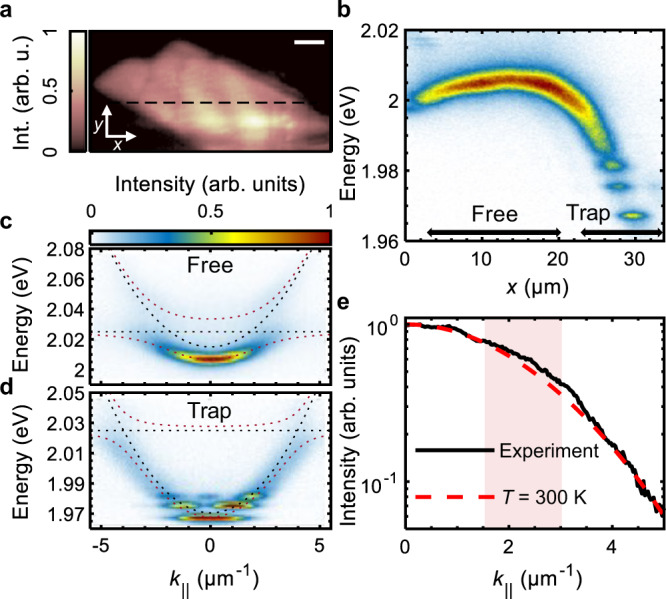# Author Correction: Motional narrowing, ballistic transport, and trapping of room-temperature exciton polaritons in an atomically-thin semiconductor

**DOI:** 10.1038/s41467-022-30383-8

**Published:** 2022-05-06

**Authors:** M. Wurdack, E. Estrecho, S. Todd, T. Yun, M. Pieczarka, S. K. Earl, J. A. Davis, C. Schneider, A. G. Truscott, E. A. Ostrovskaya

**Affiliations:** 1 ARC Centre of Excellence in Future Low-Energy Electronics Technologies and Nonlinear Physics Centre, Research School of Physics, The Australian National University, Canberra, ACT Australia; 2 Department of Experimental Physics, Wrocław University of Science and Technology, Wrocław, Poland; 3 ARC Centre of Excellence in Future Low-Energy Electronics Technologies and Centre for Quantum and Optical Science, Swinburne University of Technology, Victoria, Australia; 4 Institut für Physik, Carl von Ossietzky Universität Oldenburg, Oldenburg, Germany; 5 Laser Physics Centre, Research School of Physics, The Australian National University, Canberra, ACT Australia

**Keywords:** Polaritons, Two-dimensional materials

Correction to: *Nature Communications* 10.1038/s41467-021-25656-7, published online 10 September 2021.

The original version of this article contained an error in Fig. 2, in which the legends “Experiment” and “*T* = 300 K” in panel “e” were reversed. This has been corrected in both the PDF and HTML versions of the Article.