# Supplementary material for: Motional narrowing, ballistic transport, and trapping of room-temperature exciton polaritons in an atomically-thin semiconductor
Source: Nat Commun. 2021 Sep 10;12:5366. doi: 10.1038/s41467-021-25656-7 (PMC8433169; doi:10.1038/s41467-021-25656-7)
Supplement: Supplementary file 1 — Supplementary Information [file 41467_2021_25656_MOESM1_ESM.pdf]

# Supplementary Information:
# Motional narrowing, ballistic transport, and trapping of room-temperature exciton polaritons in an atomically-thin semiconductor

M. Wurdack,[1] E. Estrecho,[1] S. Todd,[1] T. Yun,[1] M. Pieczarka,[1,2] S. K. Earl,[3] J. A. Davis,[3] C. Schneider,[4] A. G. Truscott,[5] and E. A. Ostrovskaya[1]

[1]*ARC Centre of Excellence in Future Low-Energy Electronics Technologies and Nonlinear Physics Centre, Research School of Physics, The Australian National University, Canberra, ACT 2601, Australia*
[2]*Department of Experimental Physics, Wrocław University of Science and Technology, Wyb. Wyspiańskiego 27, 50-370 Wrocław, Poland*
[3]*ARC Centre of Excellence in Future Low-Energy Electronics Technologies and Centre for Quantum and Optical Science, Swinburne University of Technology, Victoria 3122, Australia*
[4]*Institut für Physik, Carl von Ossietzky Universität Oldenburg, Ammerländer Heerstraße 114-118, 26126 Oldenburg, Germany*
[5]*Laser Physics Centre, Research School of Physics, The Australian National University, Canberra, ACT 2601, Australia*

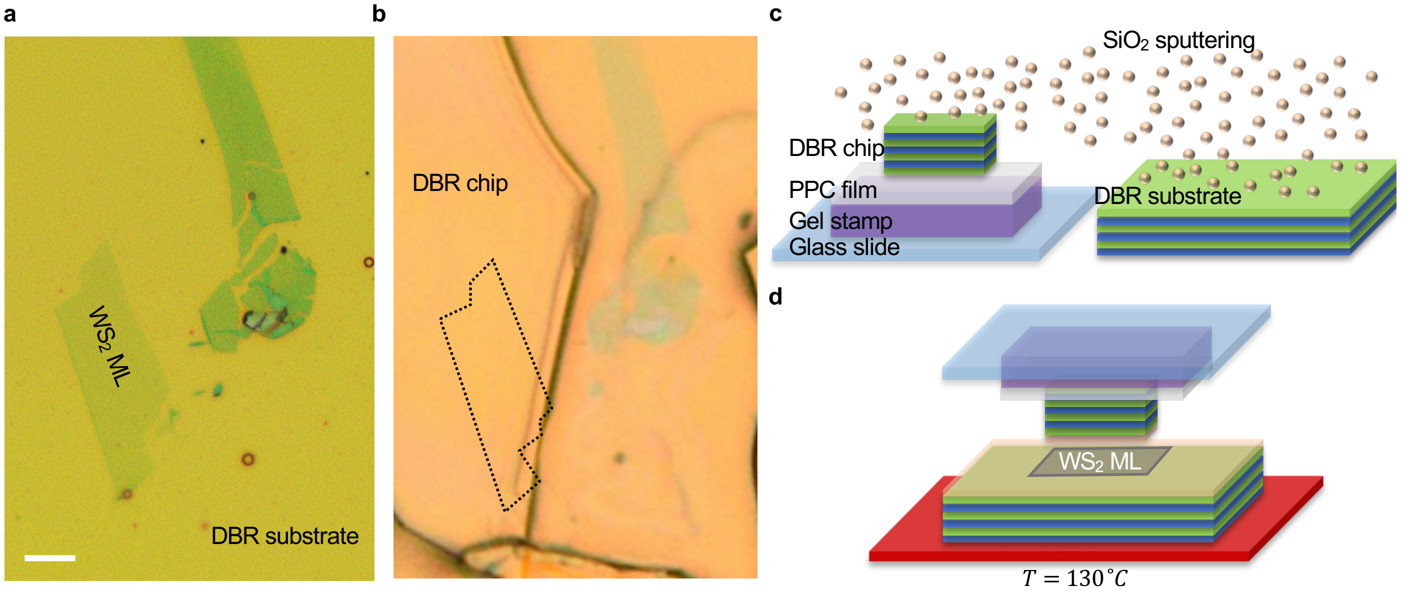


Supplementary Fig. 1. **Fabrication process.a** Microscope images of the monolayer $WS_2$ on top of a DBR-substrate, **b** Same as in panel **a**, but for a monolayer covered with a DBR chip. Scale bar = 10 $\mu$m. **c** Schematics for the fabrication process of the all-dielectric monolithic microcavity. (i) RF-magnetron sputtering of the two halves of the $\lambda/2$ $SiO_2$-spacer on top of the DBR substrate and on top of the DBR-chip placed on a polypropylene-carbonate (PPC) film with a Glass-slide/gel-stamp backbone. The DBR chip (DBR substrate) consists of 15 (17) alternating $SiO_2$ and 16 (18) $Si_3N_4$ layers with thicknesses of 105 nm and 80 nm, respectively, fabricated by plasma enhanced chemical vapour deposition (PECVD). The stop-band of the DBR chip and the DBR substrate have a central wavelength of $\lambda_C = 615$ nm. (ii) Assembly of the all-dielectric microcavity at $T = 130$ °C, with a $WS_2$ monolayer placed on the DBR substrate at an intermediate step.

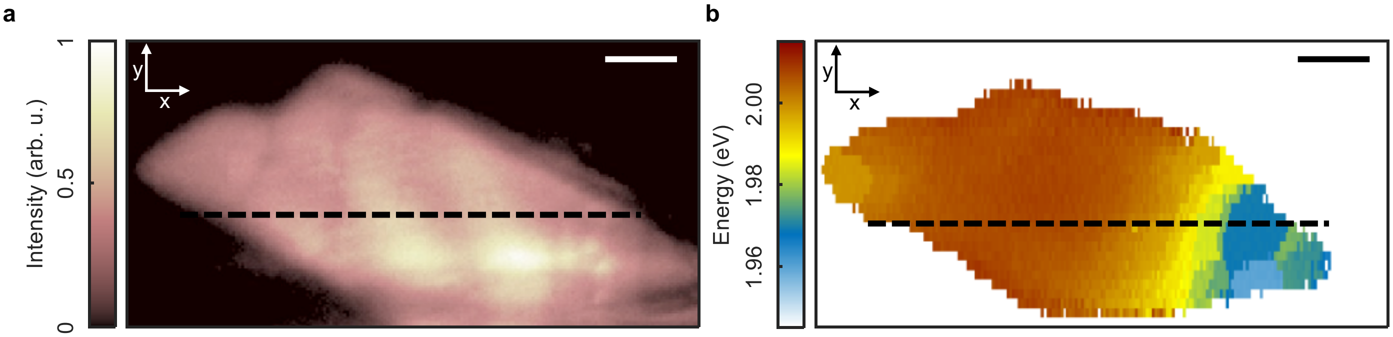


Supplementary Fig. 2. **Potential landscape of the microcavity.a** Photoluminescence map of the microcavity at the monolayer region, excited with a Nd:YAG frequency doubled continuous-wave (cw) laser at $\lambda = 532$ nm, as shown in Fig. 2a of the main text. Scale bar = 5 $\mu$m. **b** Potential landscape of the monolayer, measured at the angles of approximately zero incidence, i.e. for zero in-plane momentum, $k_{\|}$. The landscape is measured by spectral tomography, where the last lens in front the spectrometer is moved step-wise, in the direction orthogonal to the spectrometer slit (i.e., perpendicular to the dashed line of panel **a** for each recorded spectrum. Concatenating the spectra results in the energy map as shown here. Axis $x(y)$ marks the direction along (perpendicular to) the spectrometer slit.

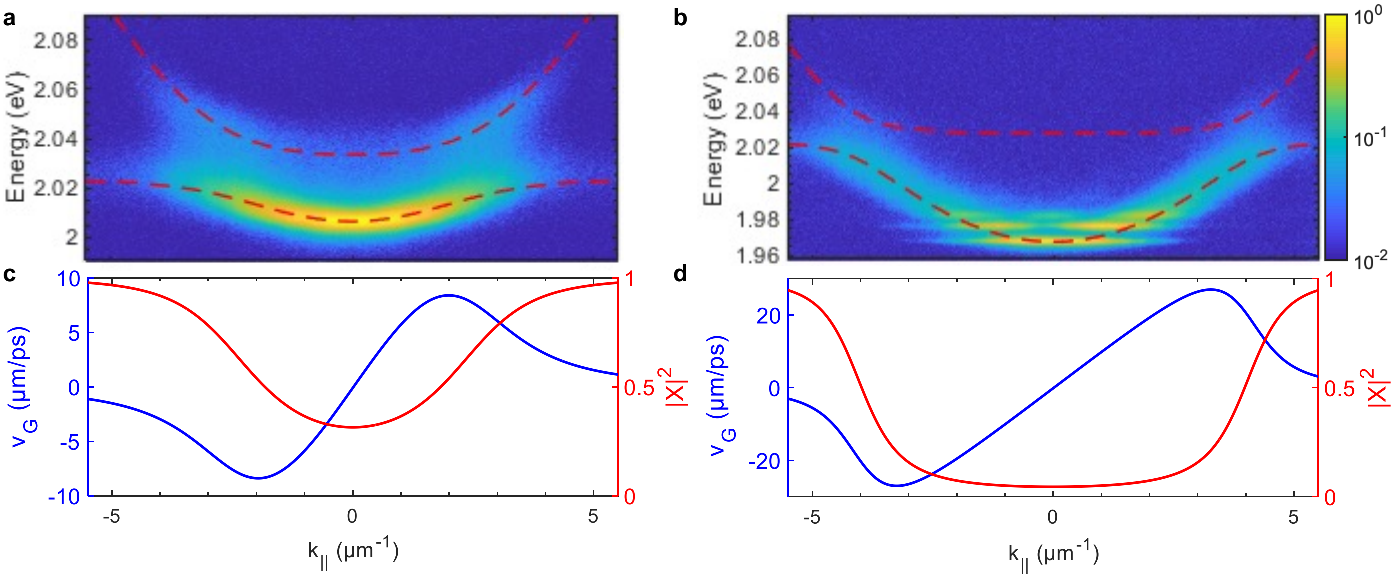


Supplementary Fig. 3. **Momentum-resolved polariton PL, group velocity, and Hopfield coefficient. a,b** Angle-resolved ($k_{\|}$-space) PL spectra of the microcavity **a** in the planar (Fig. 2c of the main text) and **b** in the trap region (Fig. 2d of the main text), showing the anti-crossing of the lower and the upper polariton branches and the emission from the upper polariton branch. The intensity scale is logarithmic. The red dashed lines represent the fitted lower and upper polariton dispersions. **c,d** Group velocity $v_{\mathrm{G}} = \delta\omega/\delta k_{\|}$ (blue) and the excitonic Hopfield coefficient (red) of the lower polariton dispersions in panels **a** and **b**, respectively. The maxima (minima) of the group velocity correspond to the inflection points of the polariton dispersion, and mark the bottleneck regimes for the free and trapped polaritons [1].

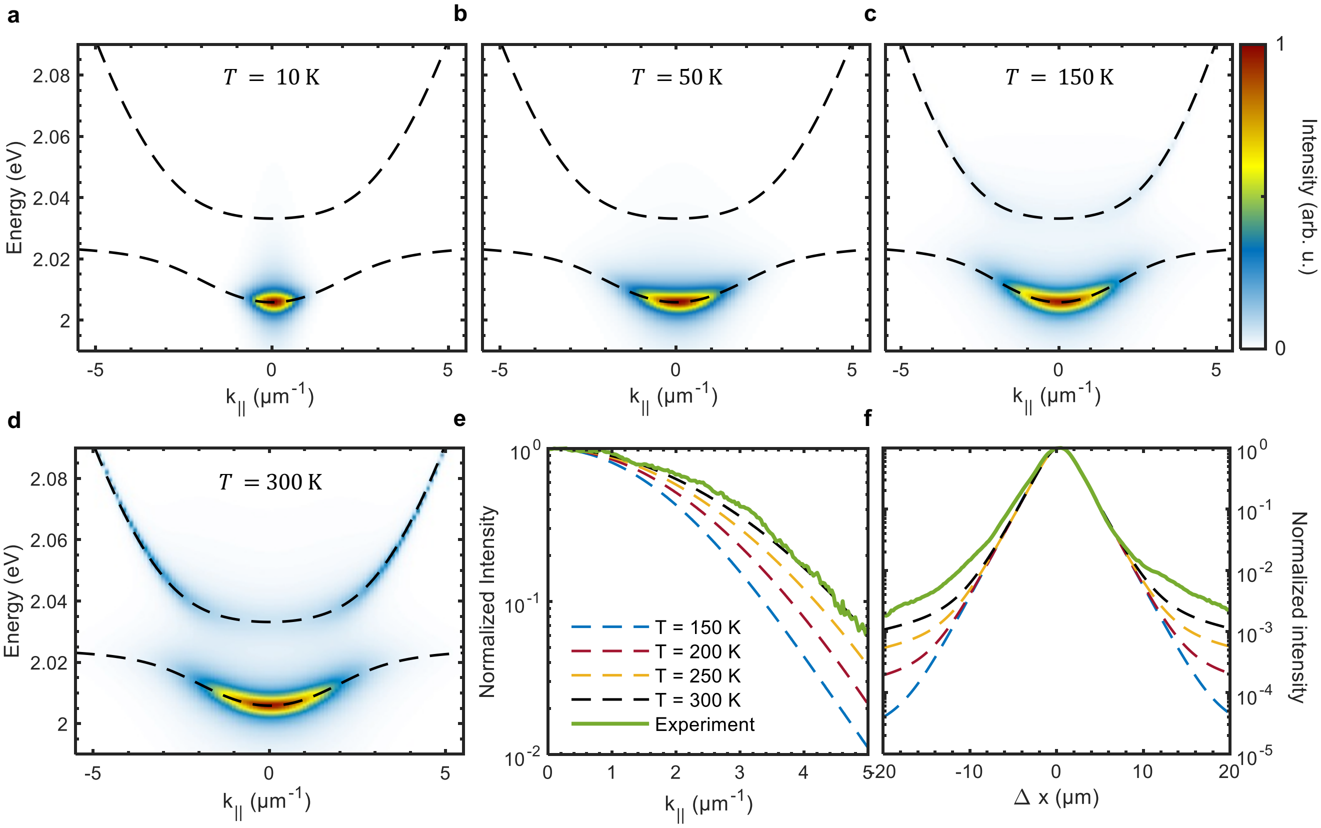


Supplementary Fig. 4. **Polariton thermalisation. a-d** Calculated momentum-resolved PL spectra for fully thermalised free polaritons with the dispersion shown in Supplementary Fig. 3a, $I(k_{||}, E) = (1 - |X(k_{||})|^2) \exp[-(E_P - E_P^0)/(k_B T)] \cdot 0.5\gamma_P \left[(E - E_P)^2 + 0.5\gamma_P^2\right]^{-1}$ [6], for **a)** $T = 10$ K, **b)** $T = 50$ K, **c)** $T = 150$ K, **d)** $T = 300$ K. **e** Experimental and theoretical intensity profiles along $k_{||}$ for the measured free polariton PL and for fully thermalised polaritons, respectively. The agreement between the theory at $T = 300$ K and the experiment indicates that the polariton gas in the experiment is almost fully thermalised at room temperature. **f** Experimental and theoretical real-space PL profile. The theoretical lines were calculated by convolving the Fourier-transformed PL profiles from panel **e** with the Gaussian excitation profile ($\mathrm{FWHM_{laser}} = 2.04\ \mu$m) and with the exponential decay of the propagating polaritons $\exp(-|\Delta x|/x_D)$. The exponential decay constant was approximated from the spatial decay of the polaritons at the inflection point of the dispersion corresponding to the group velocity extremum $x_D = \tau(v_G^{max})v_G^{max}$, with $\tau(v_G^{max})$ calculated from the linewidth of the PL at this momentum. Increasing the temperature of the polariton gas leads to increasing polariton propagation length, and approaches the measured PL profile at $T = 300$ K. Hence, room temperature promotes the ballistic expansion of the polariton gas, as shown in the main text.

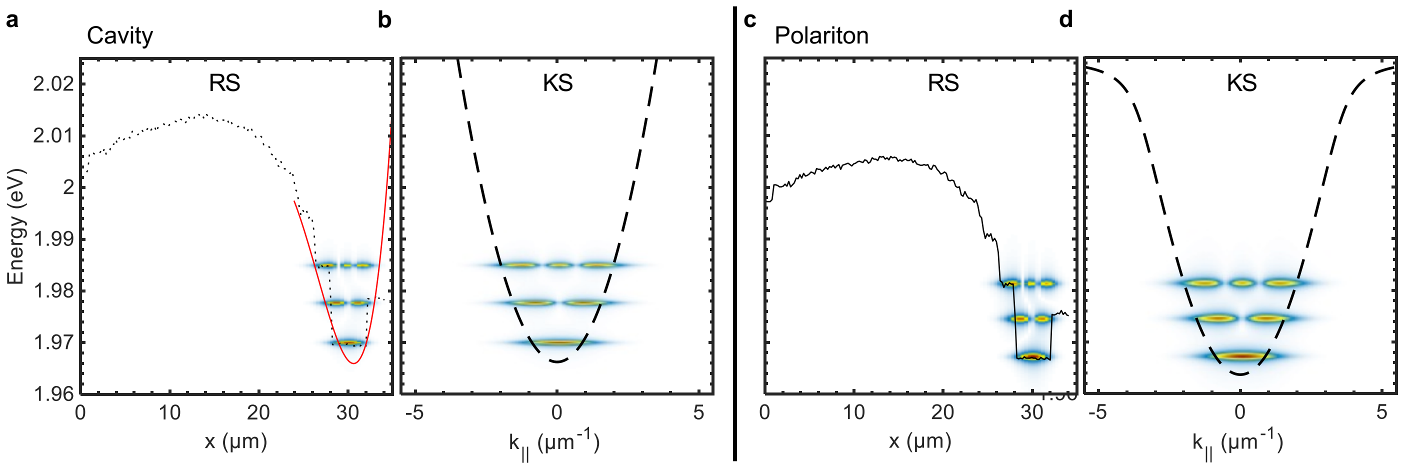


Supplementary Fig. 5. **Simulations of real-space (RS) and momentum-resolved (KS) PL spectra in the trap region.** **a,b** Calculated spectra for cavity photons. The energy of the cavity mode at $k_{||} \approx 0$ (black dotted line in panel **a**) is reconstructed from the extracted profile of the polariton emission at $k_{||} \approx 0$ (black solid line in panel **c**). The shape of the effective potential for the cavity photon in the trap region can be approximated with a polynomial function of $4^{th}$ order (red solid line in panel **a**). The energy eigenstates in this effective potential are found by solving the Schrödinger equation for a cavity photon with the effective mass $m_{\text{eff}}^{\text{C}} = 0.8 \times 10^{-5}\ m_e$ (by using the MATLAB [2] library Chebfun [3]). The deviation from the effective mass of the free cavity photons is due to the air gap in the trap region. The PL spectra of the trapped photons in real space (RS) and momentum space (KS) (Fourier transform of RS) in panels **a,b** take into account the experimental linewidth of the cavity mode (see. Supplementary Fig. 9). **c,d** PL spectra for trapped polaritons calculated using the coupled-oscillator model for the cavity photons and $WS_2$ excitons, with the photon modes from **a,b** and the experimentally determined exciton linewidth and the Rabi splitting $2\hbar\Omega \approx 25$ meV.

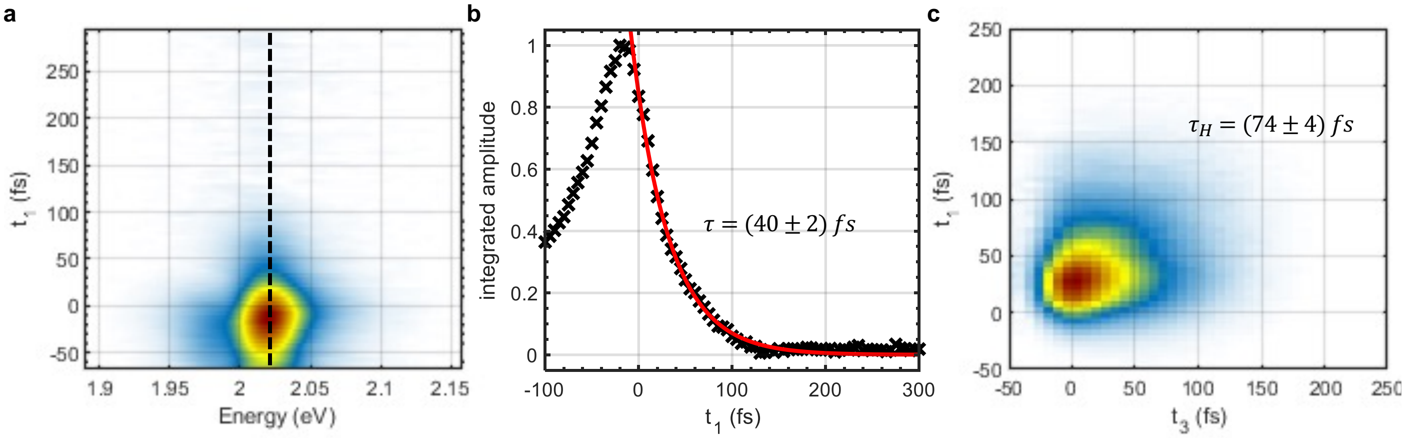


Supplementary Fig. 6. **Measurements of the dephasing and decoherence times of excitons in a monolayer $WS_2$ on $SiO_2$.** **a** Energy- and time- resolved non-rephasing data around the energy of $WS_2$ excitons measured via multidimensional coherent spectroscopy (MDCS). **b** Profile (black crosses) along the dashed line in panel **a**, fitted with a single-exponential decay function with a decay time of $\tau = (40\pm)$ fs. **c** Rephasing scan of the (MDCS) unveiling a decoherence time of $\tau = (74 \pm 4)$ fs for the $WS_2$ excitons, which is related to the homogeneous linewidth broadening.

### Supplementary Note: Multidimensional coherence spectroscopy of monolayer $WS_2$ on $SiO_2$.

The $WS_2$ exciton decoherence and dephasing times were measured using multidimensional coherent spectroscopy (MDCS), a class of four-wave mixing experiments capable of measuring homogeneous linewidths in the presence of inhomogeneous broadening [4].

The measurements were conducted using 23 fs pulses with central wavelength of 620 nm, generated by a non-colinear optical parametric amplifier (NOPA) pumped by a Yb:KGW laser amplifier. The fluence for these measurements was 1.2 $\mu$Jcm$^{-2}$ per pulse or $3.7 \times 10^{12}$ photons per $cm^2$ per pulse. Intensity dependent measurements were conducted to confirm that at this fluence exciton-exciton scattering has minimal impact on the decoherence time. A freshly exfoliated $WS_2$ monolayer was placed on a silicon substrate covered in approximately 300 nm of $SiO_2$ and imaged in situ using a telescope constructed from thin lenses. Rephasing scans, corresponding to a photon echo pulse ordering were taken at room temperature to extract the homogeneous decoherence time, while non-rephasing scans were used to determine the dephasing dynamics including the loss of macroscopic coherence due to the inhomogeneous distribution of exciton energies in addition to the homogeneous decoherence processes.

For the non-rephasing data (see Supplementary Fig. 6a), the response was spectrally integrated around the A-exciton energy and a single decaying exponential function was fitted to the signal (see Supplementary Fig. 6b). A decay time of $(40 \pm 2)$ fs as a product of exciton decoherence and dephasing, i.e. macroscopic decoherence, was determined as the average of multiple scan repeats. For the rephasing data, the fact that the inhomogeneous broadening is similar in magnitude to the homogeneous linewidth means that we cannot take the inhomogeneous limit (where the decoherence time would be twice the measured decay time) or the homogeneous limit (where the decoherence time would be equal to the decay time). To overcome this we make use of the fact that in these MDCS experiments we collect both amplitude and phase information, which allows us to Fourier transform the data from the spectral domain to the time domain, and reveal the time evolution of the signal in both $t_1$ (the delay between the first two pulses that is scanned) and $t_3$ (the Fourier transformed emission energy, corresponding to the time between third pulse and signal emission) time periods (see Supplementary Fig. 6c). From this we are able to fit the 2D data to equation [5]

$$E = E_0 \exp\left(-(t_1 + t_3)/T_2 + \sigma^2(t_3 - t_1)^2/2\right),$$

which describes the signal evolution, without having to take either limit, and removes the ambiguity. Here, $E_0$ is the electric field amplitude, $T_2$ is the homogeneous decoherence time, $\sigma$ is the width of the Gaussian inhomogeneous broadening and the oscillating part of the electric field has been left out, as we take the absolute value from the experimental results. By averaging the results of five repeated scans, and fits for data with different waiting times, we determine a decoherence time of $(74 \pm 4)$ fs. This results is in good agreement with the Lorentzian linewidth of the Voigt fit to the $WS_2$ excitons, as discussed in the main text.

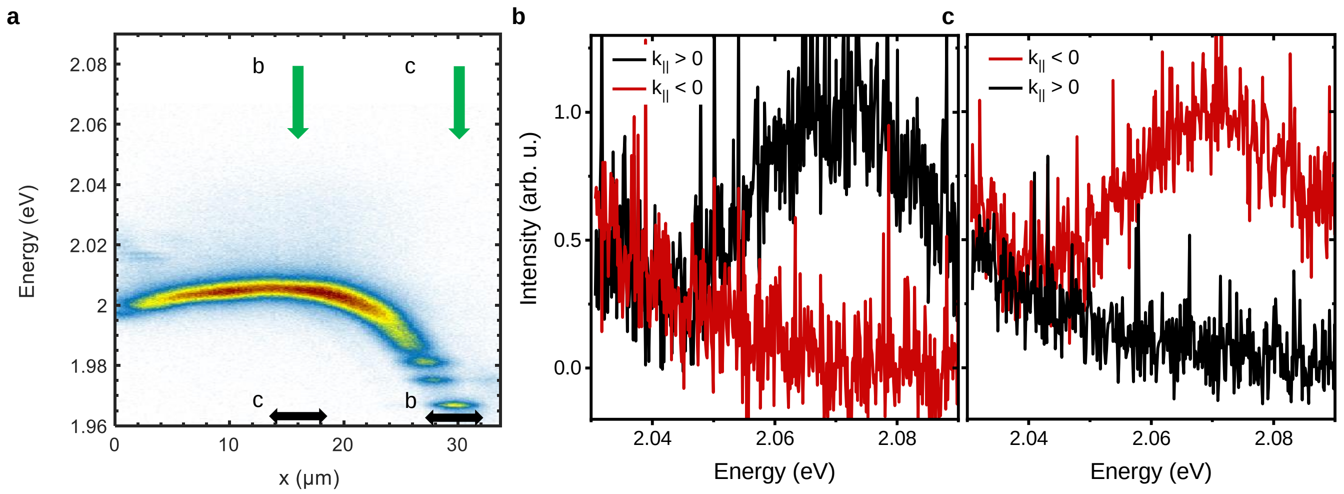


Supplementary Fig. 7. **Directional propagation of the upper polaritons.** **a** Potential landscape of the microcavity along the dashed line in Supplementary Fig. 2b. **b,c** PL-intensity of the angle-resolved PL spectra of the upper polariton branch excited at $x = 16$ $\mu$m ($x = 30$ $\mu$m) and measured at $x = 30$ $\mu$m ($x = 16$ $\mu$m). The spectra clearly show that the upper polaritons exhibit a directional flow and that the energy of the flow remains constant independently on the energy of the ground state at the excitation spot, as shown in the main text for the lower polaritons. The absence of signal at the momenta opposite to the propagation directions further confirms that backscattering on dielectric disorder can be neglected in this system.

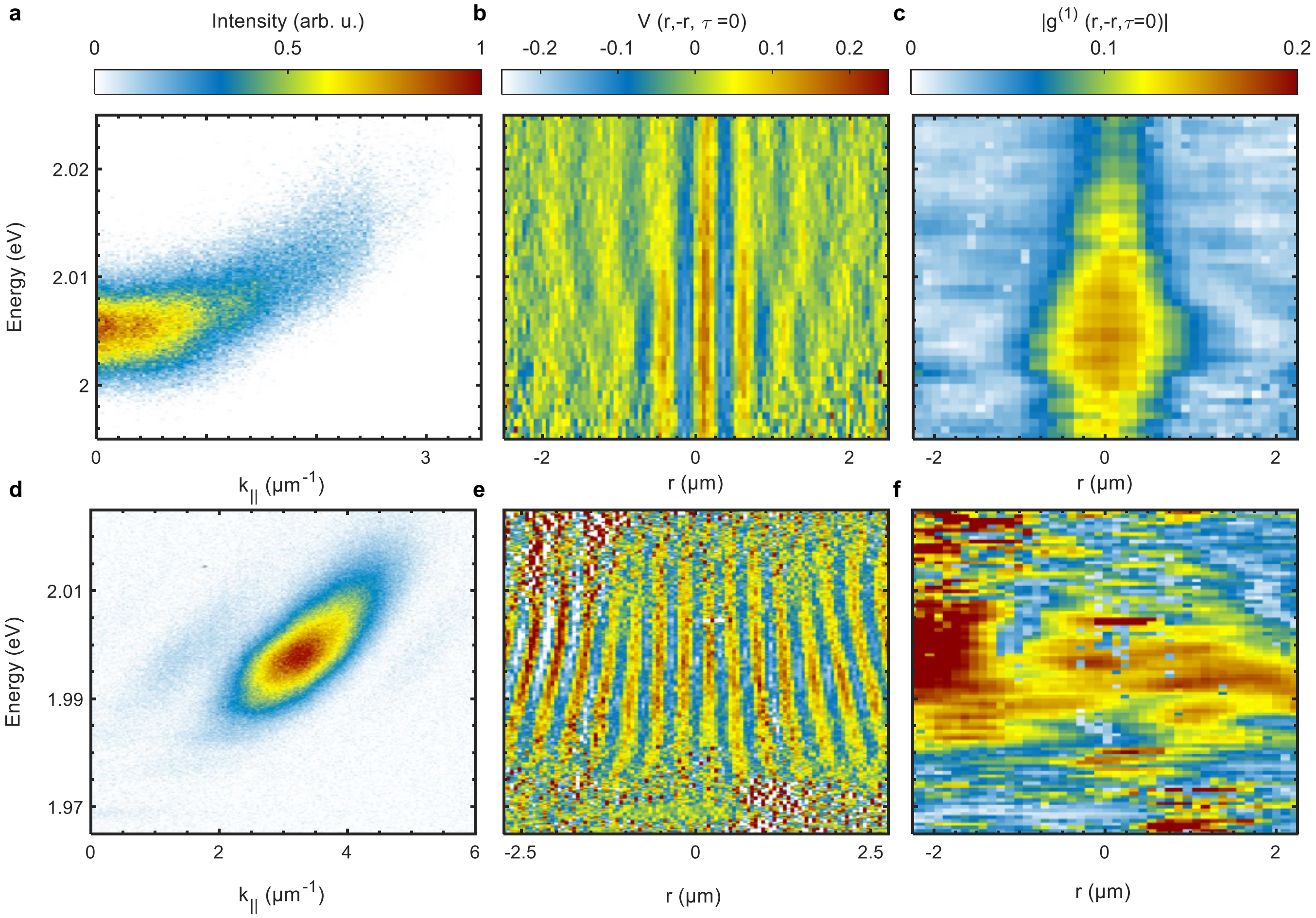


Supplementary Fig. 8. **First-order coherence measurements with the modified Michelson Interferometer on and away from the excitation spot. a,d** Density of states (DOS), **b,e** spectrally resolved interference measurement, and **c,f** extracted $|g^{(1)}|$ map of the polaritons on (a,b,c) the planar region ($x = 14$ $\mu$m), at the spot of excitation, and on (**d,e,f**) the trap region ($x = 30$ $\mu$m), next to the spot of excitation ($x = 23$ $\mu$m), respectively. The interference fringes and the $|g^{(1)}|$ maps show that macroscopic coherence is accumulated at maximum DOS. The magnitude of partial macroscopic coherence is approximately the same at the spot of excitation and away from it, exhibiting low dephasing and decoherence for the travelling polaritons.

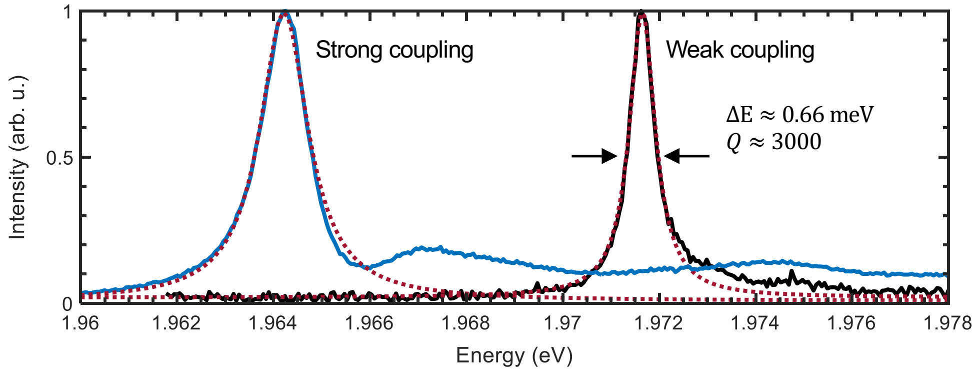


Supplementary Fig. 9. **Measurement of the microcavity quality factor.** Photoluminescence spectrum at the angles of approximately zero incidence ($k_{||} \approx 0$) in the trap region of the microcavity in the strong coupling (blue solid line) and in the weak coupling (black solid line) regime. The red dashed lines are Lorentzian fits to the data. The embedded $WS_2$ monolayer significantly degraded after the long laser exposure which resulted in the transition from the strong to the weak coupling regime. This is visible in the blueshift of the emission from $E \approx 1.964$ eV (polariton mode) to $E \approx 1.972$ eV (cavity mode) accompanied by the linewidth narrowing from 1.2 meV to 0.66 meV. The linewidth of the structure in the weak coupling regime approximately corresponds to the Q-factor of the microcavity measured to be $Q \approx 3000$. The actual Q-factor is, most likely, larger than this value due to the limited resolution of the setup, effect of spatial averaging of the emission and losses in the monolayer.

[1] Byrnes, T., Kim, N. Y. & Y. Yamamoto, Exciton-polariton condensates, *Nat. Phys.* **10**, 803-813 (2014).
[2] MATLAB 2020a, The MathWorks, Natick (2020).
[3] Driscoll, T. A., Hale, N. & Trefethen, L. N. Editors, *Chebfun Guide* (Pafnuty Publications, Oxford, 2014).
[4] Tollerud, J. A. & Davis, J. A. Coherent multi-dimensional spectroscopy: Experimental considerations, direct comparisons and new capabilities, *Prog. Quantum Electron.* **55**, 1-34 (2017).
[5] Siemens, M. E., Moody, G., Li, H., Bristow, A. D. & Cundiff, S. T. Resonance lineshapes in two-dimensional Fourier transform spectroscopy, *Opt. Express* **18**, 17 (2010).
[6] Lundt, N., Klembt, S., Cherotchenko, E., Betzold, S., Iff, O., Nalitov, A. V., Klaas, M., Dietrich, C. P., Kavokin, A. V., Höfling, S. & Schneider, C. Room-temperature Tamm-plasmon exciton-polaritons with a $WSe_2$ monolayer, *Nat. Commun.* **7**, 13328 (2016).
